# Report upon Histology and Microscopy

**Published:** 1870-10

**Authors:** Homer Judd


### REPORT UPON HISTOLOGY AND MICROSCOPY.

BY HOMER JUDD, D. D. S.

Read before the American Dental Association.

We find upon referring to the Transactions of the Association that only one written report upon this subject has been submitted since a Committee on Histology has been added to the list of standing Committees. In the absence of regular consecutive reports upon this subject, it has been deemed best by the Committee not to confine themselves strictly to the developments of the past year, thinking that it might better subserve the interest of the Association to refer briefly to some of the most important observations that have been made since our most common text books upon this subject have been written. Of all the structures which have engaged the attention of microscopists during the last few years, no one has attracted more attention than the white blood corpuscle. The comparatively small number of these elements found in the blood, the little importance hitherto attached to them, the difficulties attending their investigation, and the general ignorance existing in relation to their whole history, had for a long time sufficed to discourage any critical study of their habits, their changes or their relations to other similar bodies which were supposed, on account of their locations and their surrounding circumstances, to be genetically and functionally alien to them.

Virchow first declared that there was no morphelogical difference between the white blood corpuscle and pus corpuscle which could be discovered by the microscope.

Von Recklinghausen discovered the contractile properties of pus cells, and declared that by virtue of this property they possessed the faculty of locomotion.

The same author had also observed that similar bodies existed in normal connective tissue, and that these were also endowed with contractile properties, by which they were enabled to change their forms and even their locations; which fact having been well established, they were designated by some authors as the wandering cells.

The observations of Conheim followed, and the demonstration of the identity of the white blood corpuscle and of the pus corpuscle was made complete.

One generalization farther and lymph corpuscles, the mucous corpuscles and the inflammatory exudation corpuscle were received into the same category and recognized as belonging to the same family, probably emanating from the same source.

But the importance of these discoveries were by no means equal to others which soon followed.

The idea seems to have first occurred to Von Recklinghausen that these leucocytic elements had something to do with the organization of fibrinous exudations and of thrombi, and Virchow soon enunciated the doctrine which recognized the white blood corpuscle as the organizer of the clot in a tied blood vessel.

He attributed this function, however, to the white cells inclosed within the clot, but farther investigation seems to indicate that this was not the case, but that the process of organization was carried on only by the leucocytes furnished by the circulating blood, and which pass through the walls of the vasa vasorum to the clot, and that those which were inclosed in the clot itself were incapable of performing any vital action.

Still later observations make it appear probable that, strictly speaking, the clot itself does not become organized, but that there is organized behind the clot a mass of connective tissue which effectually seals up the vessel, and that the only action of the clot itself is the physical one of protecting the organizing mass from the influence of the blood circulating in the vessel, the organizations being carried on in the same manner as the exudations from wounds and inflammations in other locations. In reference to the way in which these organizations are brought about, it has been observed that in the exudation which takes place, leucocytes are first seen in the clear fluid, having migrated there in a few hours after the reception of a wound.

The cells change their characteristics, become much larger with large nuclei, and afterwards assume the form of spindle shaped cells, which lie side by side, and finally are wrought out into the fibrous elements of ordinary connective tissue.

These details have been principally worked out by Aufrecht by a carefully conducted series of experiments upon different animals. The microscope has also been busily engaged in endeavoring to solve the mystery which hangs over the birth and parentage of these important elements of the blood. It is generally supposed that they have their origin in the lymphatic ganglia and the spleen, and some have claimed that they are formed in the liver and even in the muscles; but absolute demonstration upon this point is still wanting.

Dr. Klien states he has seen white corpuscles multiplying in at least three different ways; by fission, by a process similar to that described by authors as budding, and by a "pinching off" process, as he calls it, in which a piece of the protoplasm is separated from the mass and is soon developed into a complete leucocyte. We can therefore readily account (says the editor of the *Lancet*) for the rapid multiplication of pus cells in abscess.

But the red corpuscles have not altogether been neglected. We learn from the *Monthly Microscopal Journal* that a paper has been submitted to the French Academy by M. M. Brechamp and Eston, in which it is claimed that they have demonstrated that the red corpuscle is not a homogeneous mass, but that it is made up of a multitude of specific elements or granules, each one of which is capable of a separate development.

They affirm that when the corpuscles are broken up and these elements set free, that they have an independent oscillatory motion; that they act like ferments, and that these micro-ferments give rise to cells like leucocytes.

We may therefore finally discover that the white corpuscles are derived first from the red globules, though this would be widely divergent from the generally accepted doctrine, which would change a white corpuscle into a red one.

The microscope has also been usefully employed in attempting to solve the many questions which have reference to the influence exerted by parasites in the production of disease. The subject is attended with so many difficulties that progress in this direction does not seem to keep pace with that witnessed in many other fields of research; but the impetus given to experimentation upon the subject by the establishment of the *Zeitschrift für parasitenkunde*, an able quarterly journal, devoted exclusively to the study of parasites and parasitic affections, it is to be hoped will yet put us in possession of many facts relating to the causes of disease that will prove of much service to scientific medicine.

In relation to the histology of the Dental tissues, we would remark that at least one important step has been gained during the last year towards the demonstration of nerve fibre in the Dentinal tubuli.

Boll, a German microscopist, had succeeded in tracing the nerve fibres of the pulp of a tooth between the cellular elements which form the periphery of the pulp to the very openings into the Dentinal tubuli.

It must be borne in mind that former observers had come to the conclusion that the nerve fibres of the pulp terminated at or about the line of junction of the membrana etonis of Rällston, and the vascular portion of the pulp, that is to say, it was held that the nerve fibril did not penetrate the cellular layer which formed the periphery of the pulp.

Beale first showed that the nerves did penetrate this layer, but as his attention was not particularly drawn to this point, he failed to trace them through this cellular layer up to the openings of the dentinal tubuli, as Boll has succeeded in doing.

Although the nerve fibres have been traced to the very openings of the tubuli, and although it has also been clearly demonstrated that they project still farther, seemingly in the very direction that would carry into the tubuli; yet it is not claimed by Boll that the demonstration is yet complete, as it might be possible that these projecting fibres might have deflected laterally at this very point, along the surface of the cellular layer, and that in the specimens showing the projecting fibres these may not have been drawn from the tubuli, as they seem to have been.

The author does not hesitate, however, to declare that he has no doubt whatever but that these nerve fibres were withdrawn from the tubuli.

The extreme care taken by this observer not to claim anything as demonstrated whilst a single link is wanting in the chain of evidence, contrasts strongly with the course pursued by the many superficial experimenters, who frequently jump at conclusions which have but very few even of the characteristics of probability, and which the most superficial examination would at once show were not worthy of a moment's attention by scientific men.

Boll comes, however, somewhat hastily to the conclusion that as he has demonstrated that there are two different kinds of processes projected from the surface of a pulp, separated from the dentine, that there are probably two different kinds of tubuli for them to pass into, one for the nerve fibre and one for the soft process of the odontoblast, or cells of the cellular layers, but he does not claim that this has been demonstrated, nor does he adduce any special argument in its favor.

We should be inclined to regard this as an entirely unauthorized assumption. We must bear in mind the fact that the processes of the odontoblast are very much larger than the finest pale nerve fibres, and as it is already shown that the nerve fibres pass up from the vascular portion of the pulp between these elongated cells, although the cells seem to be placed closely together. So we may well suppose that these fibres continue to pass up by the side of the processes of the cells which extend into the tubuli. This opinion is strengthened by considering that there is an abundance of room for these fine nerve fibres to ramify upon the membrane which doubtless lines the inner surface of the tubular wall, without interfering with the soft fibre within the tubuli.

It is true that in the illustrations furnished of these two varieties of projecting fibres the nerve fibre is shown to project at a short distance from the side of the soft cell process, but as only a small proportion of the soft fibres are drawn out of the tubuli, the greater number breaking off at the extremity of the odontoblast, this furnishes no evidence opposed to the proposition submitted above.

Many other subjects of much interest in their relations to microscopal science seemed to invite the attention of the Committee, but they did not deem it best to extend the report farther than was absolutely necessary to give a brief résumé of a few of the most important developments which have been brought out in the last few years.

